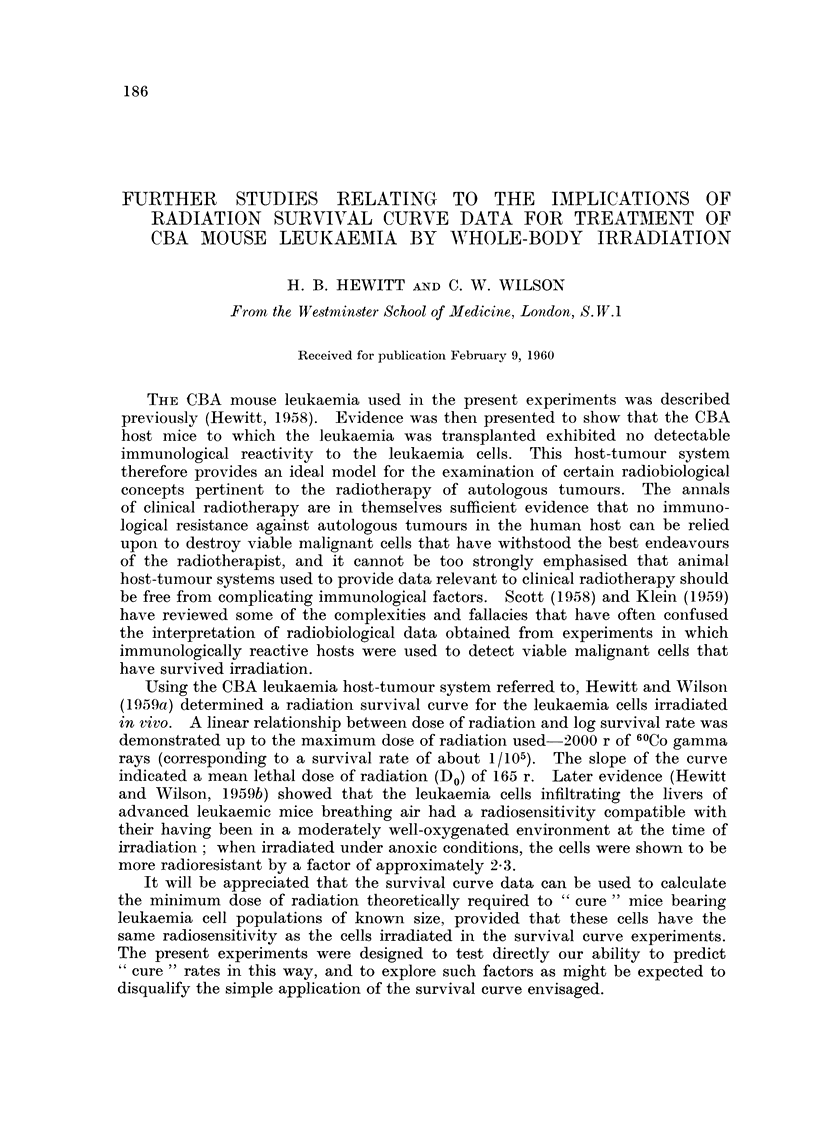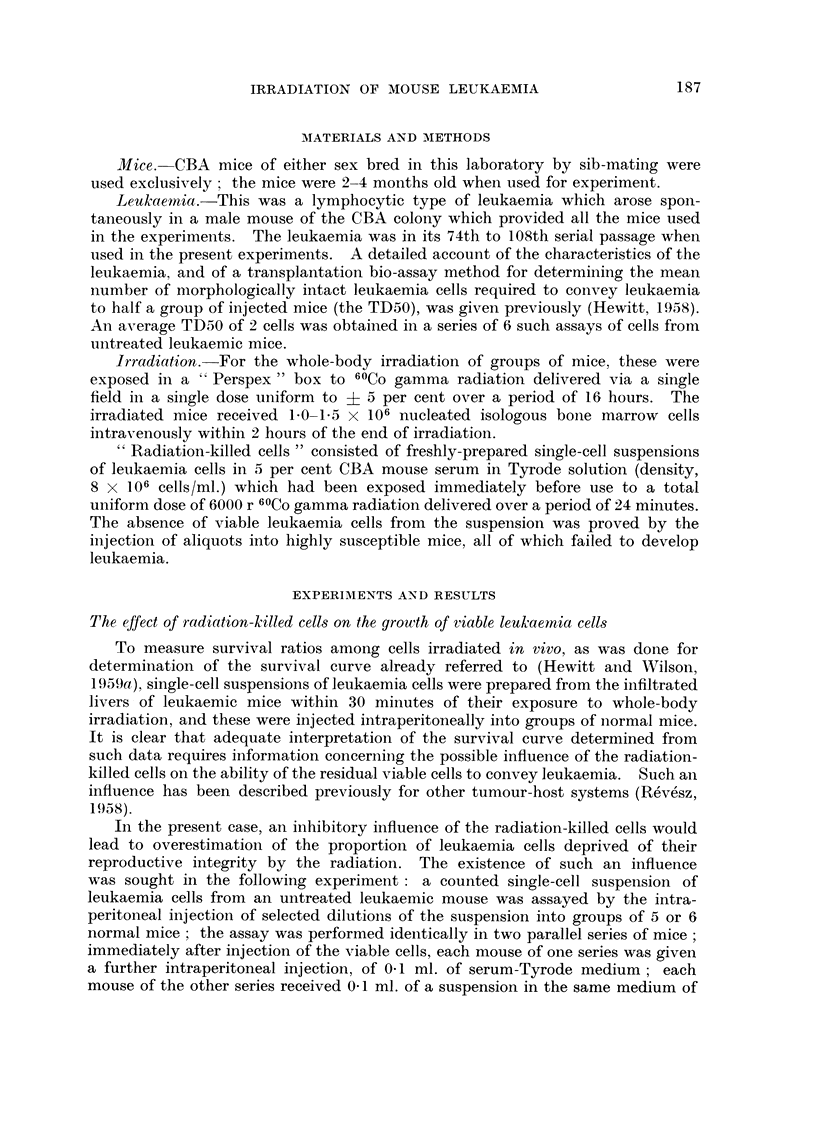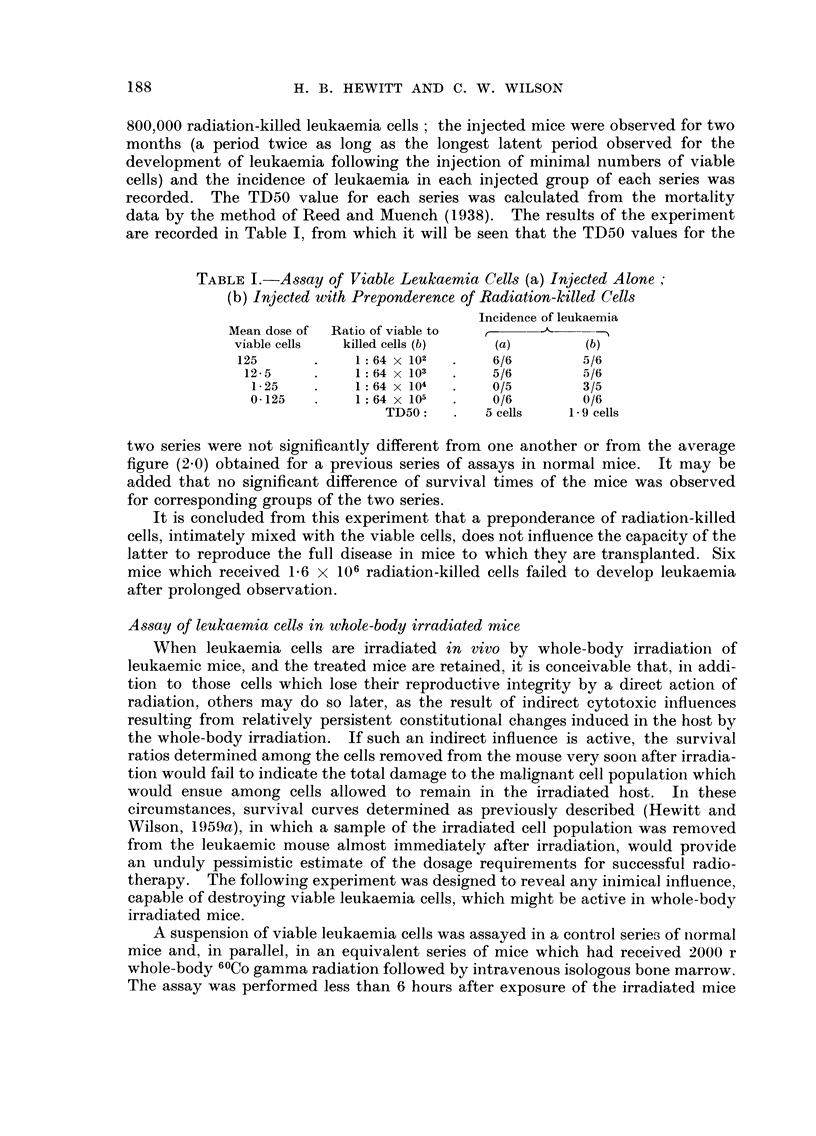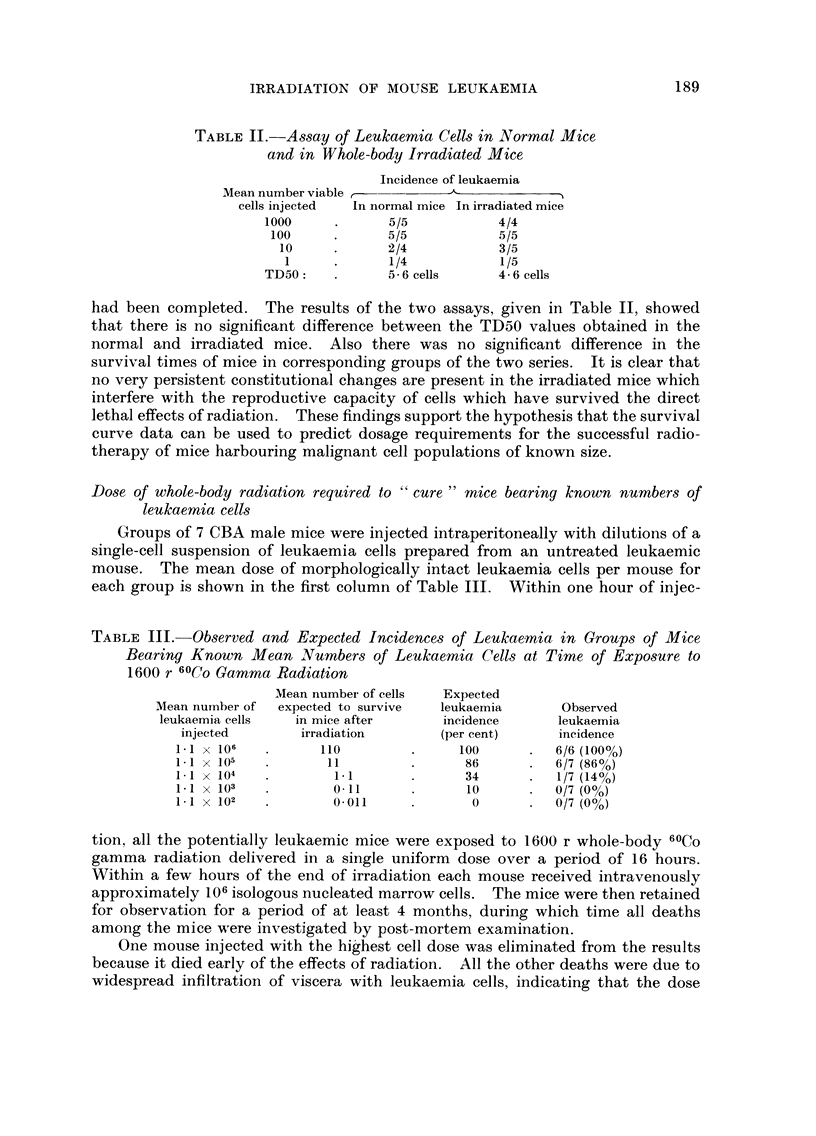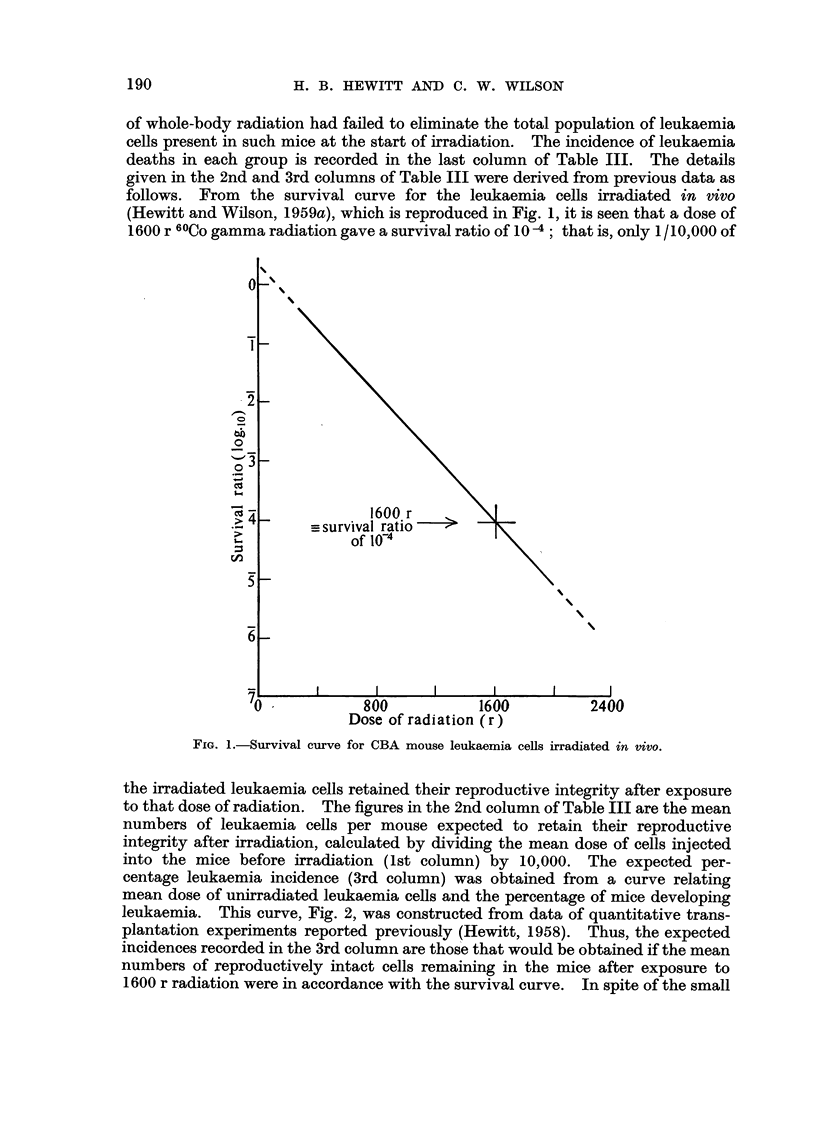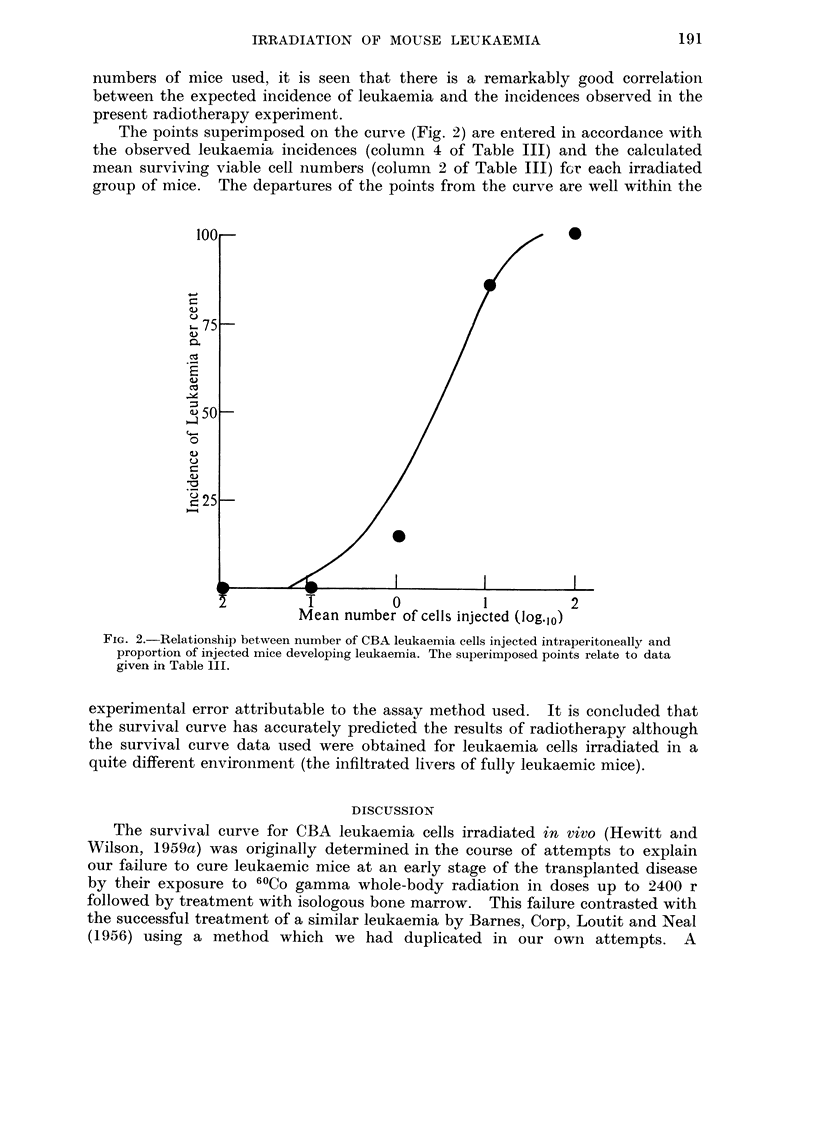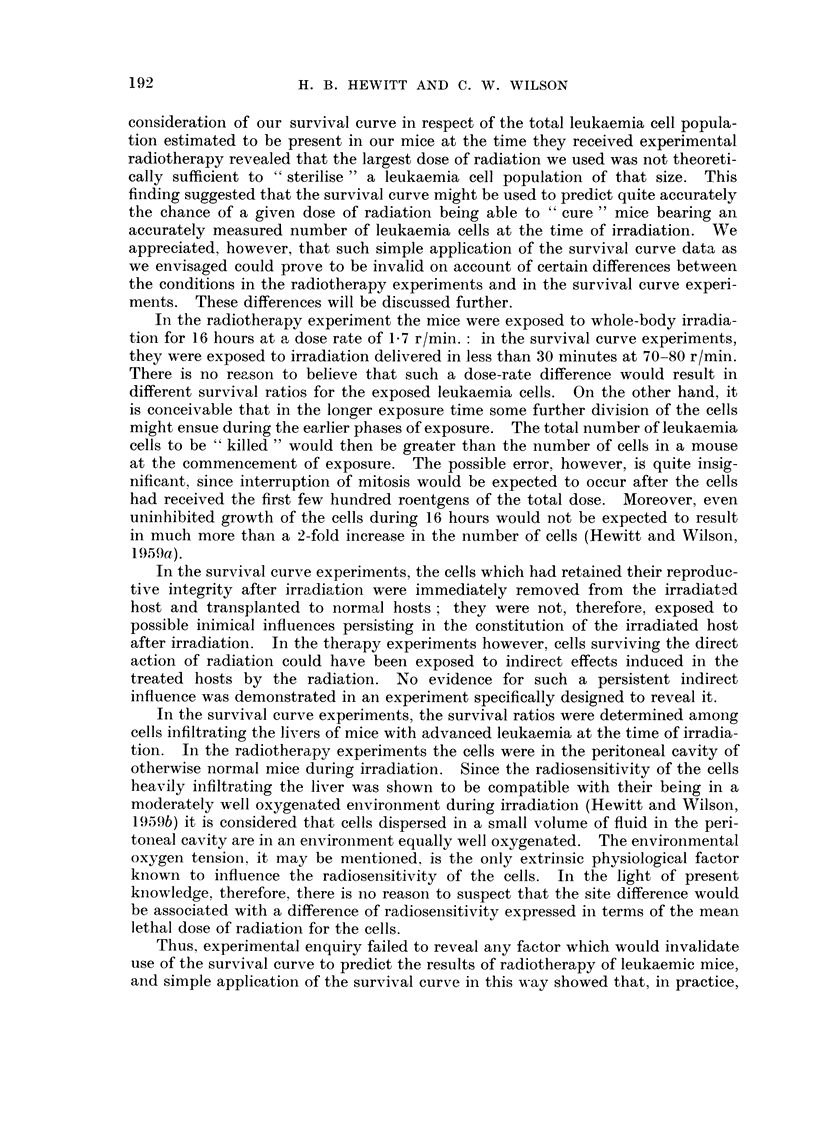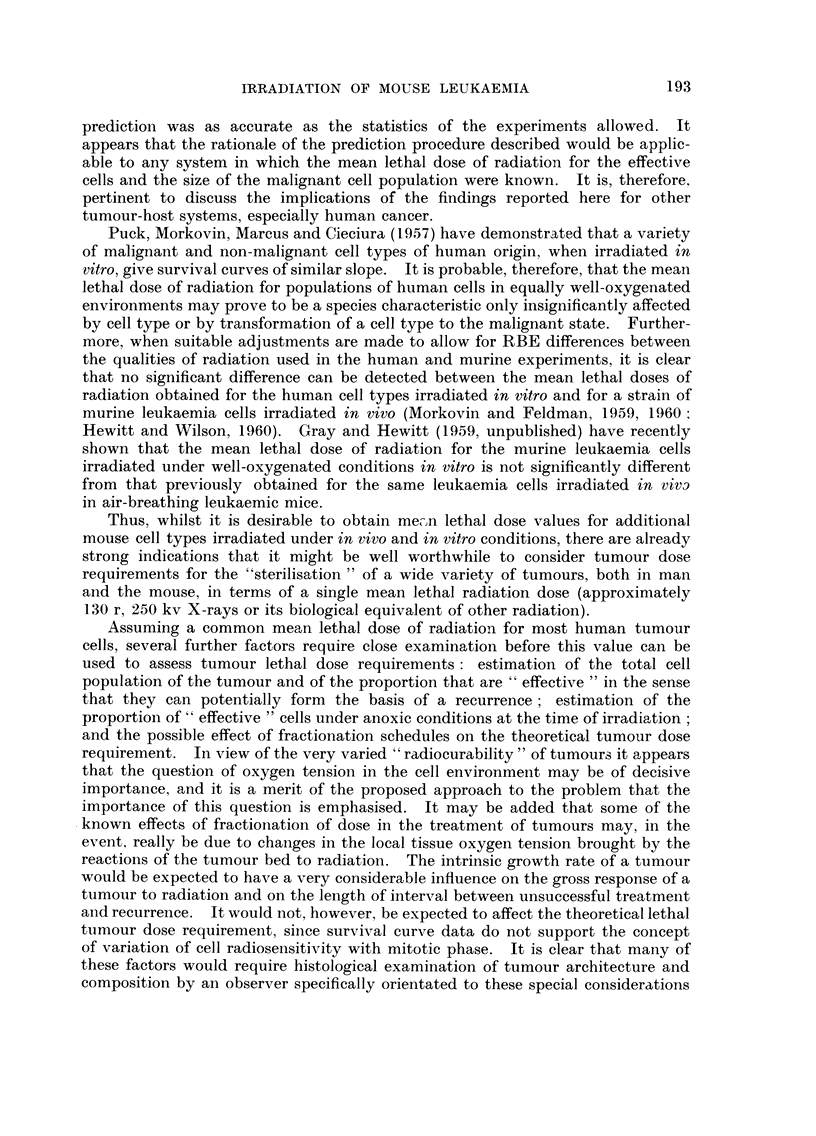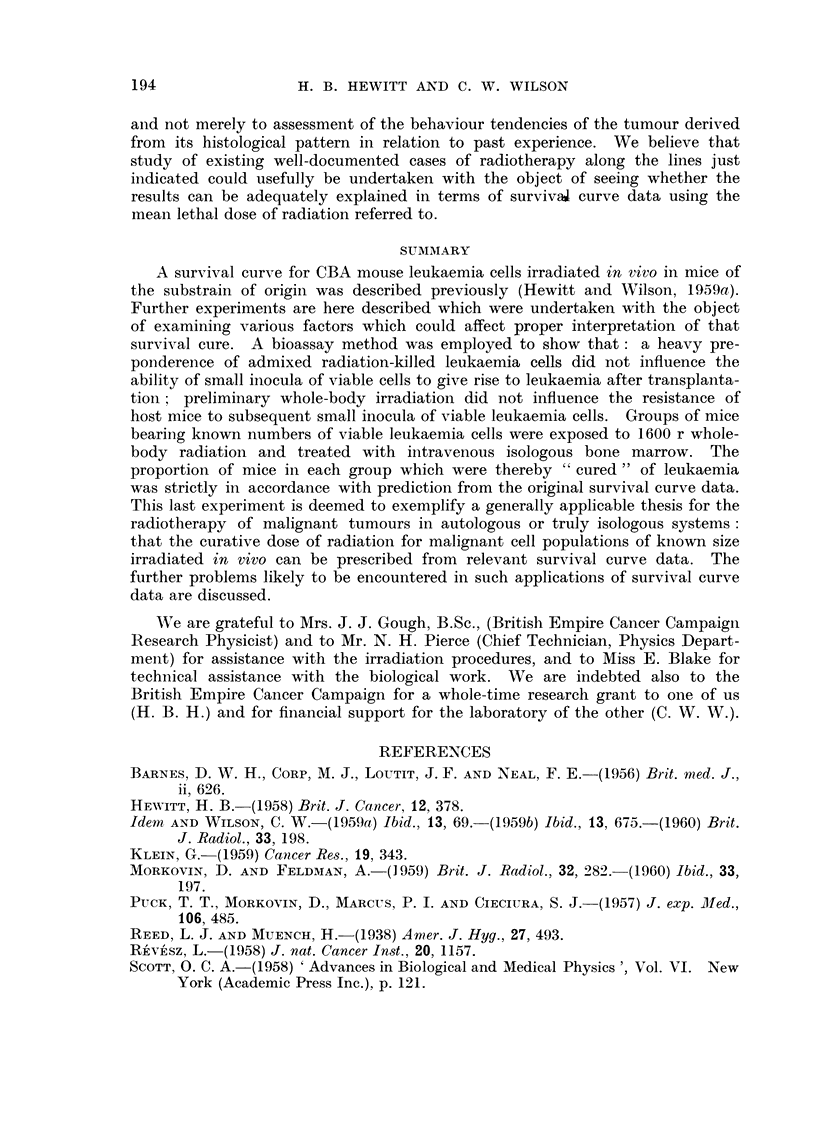# Further Studies Relating to the Implications of Radiation Survival Curve Data for Treatment of CBA Mouse Leukaemia by Whole-body Irradiation

**DOI:** 10.1038/bjc.1960.22

**Published:** 1960-06

**Authors:** H. B. Hewitt, C. W. Wilson


### FURTHER STUDIES RELATING TO THE IMPLICATIONS OF RADIATION SURVIVAL CURVE DATA FOR TREATMENT OF CBA MOUSE LEUKAEMIA BY WHOLE-BODY IRRADIATION

H. B. HEWITT AND C. W. WILSON

*From the Westminster School of Medicine, London, S.W.1*

Received for publication February 9, 1960

THE CBA mouse leukaemia used in the present experiments was described previously (Hewitt, 1958). Evidence was then presented to show that the CBA host mice to which the leukaemia was transplanted exhibited no detectable immunological reactivity to the leukaemia cells. This host-tumour system therefore provides an ideal model for the examination of certain radiobiological concepts pertinent to the radiotherapy of autologous tumours. The annals of clinical radiotherapy are in themselves sufficient evidence that no immunological resistance against autologous tumours in the human host can be relied upon to destroy viable malignant cells that have withstood the best endeavours of the radiotherapist, and it cannot be too strongly emphasised that animal host-tumour systems used to provide data relevant to clinical radiotherapy should be free from complicating immunological factors. Scott (1958) and Klein (1959) have reviewed some of the complexities and fallacies that have often confused the interpretation of radiobiological data obtained from experiments in which immunologically reactive hosts were used to detect viable malignant cells that have survived irradiation.

Using the CBA leukaemia host-tumour system referred to, Hewitt and Wilson (1959*a*) determined a radiation survival curve for the leukaemia cells irradiated *in vivo*. A linear relationship between dose of radiation and log survival rate was demonstrated up to the maximum dose of radiation used—2000 r of $^{60}$Co gamma rays (corresponding to a survival rate of about $1/10^5$). The slope of the curve indicated a mean lethal dose of radiation ($D_0$) of 165 r. Later evidence (Hewitt and Wilson, 1959*b*) showed that the leukaemia cells infiltrating the livers of advanced leukaemic mice breathing air had a radiosensitivity compatible with their having been in a moderately well-oxygenated environment at the time of irradiation ; when irradiated under anoxic conditions, the cells were shown to be more radioresistant by a factor of approximately 2·3.

It will be appreciated that the survival curve data can be used to calculate the minimum dose of radiation theoretically required to " cure " mice bearing leukaemia cell populations of known size, provided that these cells have the same radiosensitivity as the cells irradiated in the survival curve experiments. The present experiments were designed to test directly our ability to predict " cure " rates in this way, and to explore such factors as might be expected to disqualify the simple application of the survival curve envisaged.

### MATERIALS AND METHODS

*Mice.*—CBA mice of either sex bred in this laboratory by sib-mating were used exclusively ; the mice were 2–4 months old when used for experiment.

*Leukaemia.*—This was a lymphocytic type of leukaemia which arose spontaneously in a male mouse of the CBA colony which provided all the mice used in the experiments. The leukaemia was in its 74th to 108th serial passage when used in the present experiments. A detailed account of the characteristics of the leukaemia, and of a transplantation bio-assay method for determining the mean number of morphologically intact leukaemia cells required to convey leukaemia to half a group of injected mice (the TD50), was given previously (Hewitt, 1958). An average TD50 of 2 cells was obtained in a series of 6 such assays of cells from untreated leukaemic mice.

*Irradiation.*—For the whole-body irradiation of groups of mice, these were exposed in a " Perspex " box to $^{60}$Co gamma radiation delivered via a single field in a single dose uniform to $\pm$ 5 per cent over a period of 16 hours. The irradiated mice received 1·0–1·5 $\times$ $10^6$ nucleated isologous bone marrow cells intravenously within 2 hours of the end of irradiation.

" Radiation-killed cells " consisted of freshly-prepared single-cell suspensions of leukaemia cells in 5 per cent CBA mouse serum in Tyrode solution (density, 8 $\times$ $10^6$ cells/ml.) which had been exposed immediately before use to a total uniform dose of 6000 r $^{60}$Co gamma radiation delivered over a period of 24 minutes. The absence of viable leukaemia cells from the suspension was proved by the injection of aliquots into highly susceptible mice, all of which failed to develop leukaemia.

### EXPERIMENTS AND RESULTS

### *The effect of radiation-killed cells on the growth of viable leukaemia cells*

To measure survival ratios among cells irradiated *in vivo*, as was done for determination of the survival curve already referred to (Hewitt and Wilson, 1959*a*), single-cell suspensions of leukaemia cells were prepared from the infiltrated livers of leukaemic mice within 30 minutes of their exposure to whole-body irradiation, and these were injected intraperitoneally into groups of normal mice. It is clear that adequate interpretation of the survival curve determined from such data requires information concerning the possible influence of the radiation-killed cells on the ability of the residual viable cells to convey leukaemia. Such an influence has been described previously for other tumour-host systems (Révész, 1958).

In the present case, an inhibitory influence of the radiation-killed cells would lead to overestimation of the proportion of leukaemia cells deprived of their reproductive integrity by the radiation. The existence of such an influence was sought in the following experiment : a counted single-cell suspension of leukaemia cells from an untreated leukaemic mouse was assayed by the intraperitoneal injection of selected dilutions of the suspension into groups of 5 or 6 normal mice ; the assay was performed identically in two parallel series of mice ; immediately after injection of the viable cells, each mouse of one series was given a further intraperitoneal injection, of 0·1 ml. of serum-Tyrode medium ; each mouse of the other series received 0·1 ml. of a suspension in the same medium of

800,000 radiation-killed leukaemia cells ; the injected mice were observed for two months (a period twice as long as the longest latent period observed for the development of leukaemia following the injection of minimal numbers of viable cells) and the incidence of leukaemia in each injected group of each series was recorded. The TD50 value for each series was calculated from the mortality data by the method of Reed and Muench (1938). The results of the experiment are recorded in Table I, from which it will be seen that the TD50 values for the

TABLE I.—*Assay of Viable Leukaemia Cells* (a) *Injected Alone ;* (b) *Injected with Preponderence of Radiation-killed Cells*

| Mean dose of viable cells | Ratio of viable to killed cells (*b*) | Incidence of leukaemia (*a*) | Incidence of leukaemia (*b*) |
|---|---|---|---|
| 125 | $1:64\times10^2$ | 6/6 | 5/6 |
| 12·5 | $1:64\times10^3$ | 5/6 | 5/6 |
| 1·25 | $1:64\times10^4$ | 0/5 | 3/5 |
| 0·125 | $1:64\times10^5$ | 0/6 | 0/6 |
| | TD50 : | 5 cells | 1·9 cells |

two series were not significantly different from one another or from the average figure (2·0) obtained for a previous series of assays in normal mice. It may be added that no significant difference of survival times of the mice was observed for corresponding groups of the two series.

It is concluded from this experiment that a preponderance of radiation-killed cells, intimately mixed with the viable cells, does not influence the capacity of the latter to reproduce the full disease in mice to which they are transplanted. Six mice which received $1{\cdot}6\times10^6$ radiation-killed cells failed to develop leukaemia after prolonged observation.

*Assay of leukaemia cells in whole-body irradiated mice*

When leukaemia cells are irradiated *in vivo* by whole-body irradiation of leukaemic mice, and the treated mice are retained, it is conceivable that, in addition to those cells which lose their reproductive integrity by a direct action of radiation, others may do so later, as the result of indirect cytotoxic influences resulting from relatively persistent constitutional changes induced in the host by the whole-body irradiation. If such an indirect influence is active, the survival ratios determined among the cells removed from the mouse very soon after irradiation would fail to indicate the total damage to the malignant cell population which would ensue among cells allowed to remain in the irradiated host. In these circumstances, survival curves determined as previously described (Hewitt and Wilson, 1959*a*), in which a sample of the irradiated cell population was removed from the leukaemic mouse almost immediately after irradiation, would provide an unduly pessimistic estimate of the dosage requirements for successful radiotherapy. The following experiment was designed to reveal any inimical influence, capable of destroying viable leukaemia cells, which might be active in whole-body irradiated mice.

A suspension of viable leukaemia cells was assayed in a control series of normal mice and, in parallel, in an equivalent series of mice which had received 2000 r whole-body $^{60}Co$ gamma radiation followed by intravenous isologous bone marrow. The assay was performed less than 6 hours after exposure of the irradiated mice

TABLE II.—*Assay of Leukaemia Cells in Normal Mice and in Whole-body Irradiated Mice*

| Mean number viable cells injected | Incidence of leukaemia In normal mice | In irradiated mice |
|---|---|---|
| 1000 | 5/5 | 4/4 |
| 100 | 5/5 | 5/5 |
| 10 | 2/4 | 3/5 |
| 1 | 1/4 | 1/5 |
| TD50 : | 5·6 cells | 4·6 cells |

had been completed. The results of the two assays, given in Table II, showed that there is no significant difference between the TD50 values obtained in the normal and irradiated mice. Also there was no significant difference in the survival times of mice in corresponding groups of the two series. It is clear that no very persistent constitutional changes are present in the irradiated mice which interfere with the reproductive capacity of cells which have survived the direct lethal effects of radiation. These findings support the hypothesis that the survival curve data can be used to predict dosage requirements for the successful radiotherapy of mice harbouring malignant cell populations of known size.

*Dose of whole-body radiation required to " cure " mice bearing known numbers of leukaemia cells*

Groups of 7 CBA male mice were injected intraperitoneally with dilutions of a single-cell suspension of leukaemia cells prepared from an untreated leukaemic mouse. The mean dose of morphologically intact leukaemia cells per mouse for each group is shown in the first column of Table III. Within one hour of injec-

TABLE III.—*Observed and Expected Incidences of Leukaemia in Groups of Mice Bearing Known Mean Numbers of Leukaemia Cells at Time of Exposure to 1600 r $^{60}Co$ Gamma Radiation*

| Mean number of leukaemia cells injected | Mean number of cells expected to survive in mice after irradiation | Expected leukaemia incidence (per cent) | Observed leukaemia incidence |
|---|---|---|---|
| $1·1 \times 10^6$ | 110 | 100 | 6/6 (100%) |
| $1·1 \times 10^5$ | 11 | 86 | 6/7 (86%) |
| $1·1 \times 10^4$ | 1·1 | 34 | 1/7 (14%) |
| $1·1 \times 10^3$ | 0·11 | 10 | 0/7 (0%) |
| $1·1 \times 10^2$ | 0·011 | 0 | 0/7 (0%) |

tion, all the potentially leukaemic mice were exposed to 1600 r whole-body $^{60}Co$ gamma radiation delivered in a single uniform dose over a period of 16 hours. Within a few hours of the end of irradiation each mouse received intravenously approximately $10^6$ isologous nucleated marrow cells. The mice were then retained for observation for a period of at least 4 months, during which time all deaths among the mice were investigated by post-mortem examination.

One mouse injected with the highest cell dose was eliminated from the results because it died early of the effects of radiation. All the other deaths were due to widespread infiltration of viscera with leukaemia cells, indicating that the dose of whole-body radiation had failed to eliminate the total population of leukaemia cells present in such mice at the start of irradiation. The incidence of leukaemia deaths in each group is recorded in the last column of Table III. The details given in the 2nd and 3rd columns of Table III were derived from previous data as follows. From the survival curve for the leukaemia cells irradiated *in vivo* (Hewitt and Wilson, 1959*a*), which is reproduced in Fig. 1, it is seen that a dose of 1600 r $^{60}Co$ gamma radiation gave a survival ratio of $10^{-4}$ ; that is, only 1/10,000 of

FIG. 1.—Survival curve for CBA mouse leukaemia cells irradiated *in vivo*.

the irradiated leukaemia cells retained their reproductive integrity after exposure to that dose of radiation. The figures in the 2nd column of Table III are the mean numbers of leukaemia cells per mouse expected to retain their reproductive integrity after irradiation, calculated by dividing the mean dose of cells injected into the mice before irradiation (1st column) by 10,000. The expected percentage leukaemia incidence (3rd column) was obtained from a curve relating mean dose of unirradiated leukaemia cells and the percentage of mice developing leukaemia. This curve, Fig. 2, was constructed from data of quantitative transplantation experiments reported previously (Hewitt, 1958). Thus, the expected incidences recorded in the 3rd column are those that would be obtained if the mean numbers of reproductively intact cells remaining in the mice after exposure to 1600 r radiation were in accordance with the survival curve. In spite of the small numbers of mice used, it is seen that there is a remarkably good correlation between the expected incidence of leukaemia and the incidences observed in the present radiotherapy experiment.

The points superimposed on the curve (Fig. 2) are entered in accordance with the observed leukaemia incidences (column 4 of Table III) and the calculated mean surviving viable cell numbers (column 2 of Table III) for each irradiated group of mice. The departures of the points from the curve are well within the

FIG. 2.—Relationship between number of CBA leukaemia cells injected intraperitoneally and proportion of injected mice developing leukaemia. The superimposed points relate to data given in Table III.

experimental error attributable to the assay method used. It is concluded that the survival curve has accurately predicted the results of radiotherapy although the survival curve data used were obtained for leukaemia cells irradiated in a quite different environment (the infiltrated livers of fully leukaemic mice).

### DISCUSSION

The survival curve for CBA leukaemia cells irradiated *in vivo* (Hewitt and Wilson, 1959*a*) was originally determined in the course of attempts to explain our failure to cure leukaemic mice at an early stage of the transplanted disease by their exposure to $^{60}Co$ gamma whole-body radiation in doses up to 2400 r followed by treatment with isologous bone marrow. This failure contrasted with the successful treatment of a similar leukaemia by Barnes, Corp, Loutit and Neal (1956) using a method which we had duplicated in our own attempts. A

consideration of our survival curve in respect of the total leukaemia cell population estimated to be present in our mice at the time they received experimental radiotherapy revealed that the largest dose of radiation we used was not theoretically sufficient to " sterilise " a leukaemia cell population of that size. This finding suggested that the survival curve might be used to predict quite accurately the chance of a given dose of radiation being able to " cure " mice bearing an accurately measured number of leukaemia cells at the time of irradiation. We appreciated, however, that such simple application of the survival curve data as we envisaged could prove to be invalid on account of certain differences between the conditions in the radiotherapy experiments and in the survival curve experiments. These differences will be discussed further.

In the radiotherapy experiment the mice were exposed to whole-body irradiation for 16 hours at a dose rate of 1·7 r/min. : in the survival curve experiments, they were exposed to irradiation delivered in less than 30 minutes at 70–80 r/min. There is no reason to believe that such a dose-rate difference would result in different survival ratios for the exposed leukaemia cells. On the other hand, it is conceivable that in the longer exposure time some further division of the cells might ensue during the earlier phases of exposure. The total number of leukaemia cells to be " killed " would then be greater than the number of cells in a mouse at the commencement of exposure. The possible error, however, is quite insignificant, since interruption of mitosis would be expected to occur after the cells had received the first few hundred roentgens of the total dose. Moreover, even uninhibited growth of the cells during 16 hours would not be expected to result in much more than a 2-fold increase in the number of cells (Hewitt and Wilson, 1959*a*).

In the survival curve experiments, the cells which had retained their reproductive integrity after irradiation were immediately removed from the irradiated host and transplanted to normal hosts ; they were not, therefore, exposed to possible inimical influences persisting in the constitution of the irradiated host after irradiation. In the therapy experiments however, cells surviving the direct action of radiation could have been exposed to indirect effects induced in the treated hosts by the radiation. No evidence for such a persistent indirect influence was demonstrated in an experiment specifically designed to reveal it.

In the survival curve experiments, the survival ratios were determined among cells infiltrating the livers of mice with advanced leukaemia at the time of irradiation. In the radiotherapy experiments the cells were in the peritoneal cavity of otherwise normal mice during irradiation. Since the radiosensitivity of the cells heavily infiltrating the liver was shown to be compatible with their being in a moderately well oxygenated environment during irradiation (Hewitt and Wilson, 1959*b*) it is considered that cells dispersed in a small volume of fluid in the peritoneal cavity are in an environment equally well oxygenated. The environmental oxygen tension, it may be mentioned, is the only extrinsic physiological factor known to influence the radiosensitivity of the cells. In the light of present knowledge, therefore, there is no reason to suspect that the site difference would be associated with a difference of radiosensitivity expressed in terms of the mean lethal dose of radiation for the cells.

Thus, experimental enquiry failed to reveal any factor which would invalidate use of the survival curve to predict the results of radiotherapy of leukaemic mice, and simple application of the survival curve in this way showed that, in practice, prediction was as accurate as the statistics of the experiments allowed. It appears that the rationale of the prediction procedure described would be applicable to any system in which the mean lethal dose of radiation for the effective cells and the size of the malignant cell population were known. It is, therefore, pertinent to discuss the implications of the findings reported here for other tumour-host systems, especially human cancer.

Puck, Morkovin, Marcus and Cieciura (1957) have demonstrated that a variety of malignant and non-malignant cell types of human origin, when irradiated *in vitro*, give survival curves of similar slope. It is probable, therefore, that the mean lethal dose of radiation for populations of human cells in equally well-oxygenated environments may prove to be a species characteristic only insignificantly affected by cell type or by transformation of a cell type to the malignant state. Furthermore, when suitable adjustments are made to allow for RBE differences between the qualities of radiation used in the human and murine experiments, it is clear that no significant difference can be detected between the mean lethal doses of radiation obtained for the human cell types irradiated *in vitro* and for a strain of murine leukaemia cells irradiated *in vivo* (Morkovin and Feldman, 1959, 1960; Hewitt and Wilson, 1960). Gray and Hewitt (1959, unpublished) have recently shown that the mean lethal dose of radiation for the murine leukaemia cells irradiated under well-oxygenated conditions *in vitro* is not significantly different from that previously obtained for the same leukaemia cells irradiated *in vivo* in air-breathing leukaemic mice.

Thus, whilst it is desirable to obtain mean lethal dose values for additional mouse cell types irradiated under *in vivo* and *in vitro* conditions, there are already strong indications that it might be well worthwhile to consider tumour dose requirements for the "sterilisation" of a wide variety of tumours, both in man and the mouse, in terms of a single mean lethal radiation dose (approximately 130 r, 250 kv X-rays or its biological equivalent of other radiation).

Assuming a common mean lethal dose of radiation for most human tumour cells, several further factors require close examination before this value can be used to assess tumour lethal dose requirements: estimation of the total cell population of the tumour and of the proportion that are "effective" in the sense that they can potentially form the basis of a recurrence; estimation of the proportion of "effective" cells under anoxic conditions at the time of irradiation; and the possible effect of fractionation schedules on the theoretical tumour dose requirement. In view of the very varied "radiocurability" of tumours it appears that the question of oxygen tension in the cell environment may be of decisive importance, and it is a merit of the proposed approach to the problem that the importance of this question is emphasised. It may be added that some of the known effects of fractionation of dose in the treatment of tumours may, in the event, really be due to changes in the local tissue oxygen tension brought by the reactions of the tumour bed to radiation. The intrinsic growth rate of a tumour would be expected to have a very considerable influence on the gross response of a tumour to radiation and on the length of interval between unsuccessful treatment and recurrence. It would not, however, be expected to affect the theoretical lethal tumour dose requirement, since survival curve data do not support the concept of variation of cell radiosensitivity with mitotic phase. It is clear that many of these factors would require histological examination of tumour architecture and composition by an observer specifically orientated to these special considerations and not merely to assessment of the behaviour tendencies of the tumour derived from its histological pattern in relation to past experience. We believe that study of existing well-documented cases of radiotherapy along the lines just indicated could usefully be undertaken with the object of seeing whether the results can be adequately explained in terms of survival curve data using the mean lethal dose of radiation referred to.

### SUMMARY

A survival curve for CBA mouse leukaemia cells irradiated *in vivo* in mice of the substrain of origin was described previously (Hewitt and Wilson, 1959*a*). Further experiments are here described which were undertaken with the object of examining various factors which could affect proper interpretation of that survival cure. A bioassay method was employed to show that: a heavy preponderence of admixed radiation-killed leukaemia cells did not influence the ability of small inocula of viable cells to give rise to leukaemia after transplantation; preliminary whole-body irradiation did not influence the resistance of host mice to subsequent small inocula of viable leukaemia cells. Groups of mice bearing known numbers of viable leukaemia cells were exposed to 1600 r whole-body radiation and treated with intravenous isologous bone marrow. The proportion of mice in each group which were thereby "cured" of leukaemia was strictly in accordance with prediction from the original survival curve data. This last experiment is deemed to exemplify a generally applicable thesis for the radiotherapy of malignant tumours in autologous or truly isologous systems: that the curative dose of radiation for malignant cell populations of known size irradiated *in vivo* can be prescribed from relevant survival curve data. The further problems likely to be encountered in such applications of survival curve data are discussed.

We are grateful to Mrs. J. J. Gough, B.Sc., (British Empire Cancer Campaign Research Physicist) and to Mr. N. H. Pierce (Chief Technician, Physics Department) for assistance with the irradiation procedures, and to Miss E. Blake for technical assistance with the biological work. We are indebted also to the British Empire Cancer Campaign for a whole-time research grant to one of us (H. B. H.) and for financial support for the laboratory of the other (C. W. W.).

### REFERENCES

Barnes, D. W. H., Corp, M. J., Loutit, J. F. and Neal, F. E.—(1956) *Brit. med. J.*, ii, 626.

Hewitt, H. B.—(1958) *Brit. J. Cancer*, **12**, 378.

*Idem* and Wilson, C. W.—(1959*a*) *Ibid.*, **13**, 69.—(1959*b*) *Ibid.*, **13**, 675.—(1960) *Brit. J. Radiol.*, **33**, 198.

Klein, G.—(1959) *Cancer Res.*, **19**, 343.

Morkovin, D. and Feldman, A.—(1959) *Brit. J. Radiol.*, **32**, 282.—(1960) *Ibid.*, **33**, 197.

Puck, T. T., Morkovin, D., Marcus, P. I. and Cieciura, S. J.—(1957) *J. exp. Med.*, **106**, 485.

Reed, L. J. and Muench, H.—(1938) *Amer. J. Hyg.*, **27**, 493.

Révész, L.—(1958) *J. nat. Cancer Inst.*, **20**, 1157.

Scott, O. C. A.—(1958) 'Advances in Biological and Medical Physics', Vol. VI. New York (Academic Press Inc.), p. 121.